# How causal is “circular causality”?

**DOI:** 10.3389/fnetp.2026.1858616

**Published:** 2026-06-22

**Authors:** Katrin Schmietendorf

**Affiliations:** Center for Data Science and Complexity, University of Münster, Münster, Germany

**Keywords:** anti-reductionism, complex systems, downward causation, emergence, network physiology, reductionism, self-organisation, synergetics

## Abstract

Network physiology addresses global physiological states that arise self-organized through the interactions among the individual components of the human organism and is therefore intimately linked to Synergetics with its central concepts of order parameters, enslavement, and circular causality. Explanations of self-organization phenomena invoking these concepts exhibit a distinctive explanatory power and scientific fruitfulness across disciplines, in part due to their appeal to downward causation. Downward causation is not only actively discussed in philosophy and philosophy of science, particularly in debates on reductionism and emergence, but is also attracting considerable interest in scientific research on complex systems. However, at least when understood in ontological terms, it remains a philosophically delicate and contested notion.

## 1 Introduction

While traditional approaches in physiology and medicine are reductionist in the sense that they consider physiological systems as isolated, network physiology conceptualizes the human organism as an integrated, multi-component network. From this viewpoint, it seeks to investigate and explain how structural organization and dynamic interactions across multiple levels and spatiotemporal scales yield global physiological states and behaviors (Ivanov 2021). Often, this involves inter-level causal dependencies. For example, sleep states or attention influence neuronal firings and *vice versa* (Glaze and Bahar, 2021; Fries et al., 2001; Fries, 2005), psychophysiological stress alters immune cell activity (Haykin and Rolls, 2021; Feng et al., 2025) and respiratory patterns modulate the rhythmic dynamics of heart cells (Ashad and Feldman, 2020; Garcia et al., 2013). In particular, appeals to causal influence from the macro-level to its constituting micro-level are philosophically intriguing because they raise the question whether such descriptions are not only explanatorily and methodologically fruitful, but also ontologically tenable, permitting deeper claims about the structure of reality in the domains under consideration. The question of causality across scales, however, is not confined to philosophical reflection, but is being actively addressed from within or motivated by the empirical sciences themselves (Potter and Mitchell, 2025; Bechtel, 2017; Auletta et al., 2007; Pezzulo and Levin, 2016), as particularly exemplified by recent works on causal emergence (Yuan et al., 2024; Hoel, 2026).

Network physiology draws on a variety of theoretical and conceptual frameworks, methods, and analytical tools, including nonlinear dynamics and complex systems theory, self-organization, time-series analysis, information-theoretic approaches, network science and graph theory, methods from statistical physics, multiscale modeling, and causal analysis (Ivanov et al., 2016; Ivanov 2021; Schöll, 2022a). Among these approaches, the theory of Synergetics is particularly illuminating with respect to the interpretation of inter-level causal relations, since its characteristic terminology of circular causality and of macroscopic order parameters that enslave their constituents provides a natural point of contact with the philosophical debate.

Synergetics explains an impressively wide range of self-organizing phenomena in terms of a few universal principles. Originating in physics, with coherent laser radiation and Bénard cells as paradigms, it has developed into an integrative theoretical framework (Haken, 1985) with applications ranging from chemistry and biology (Haken and Wunderlin, 1987; Haken, 1999; Plath, 2016) to movement coordination (Fuchs and Kelso, 2018), cognition and neuroscience (Haken and Stadler, 1990; Friedrich and Uhl, 1992; Tass and Haken, 1996), psychology (Schiepek and Haken, 2010; Schiepek et al., 2016; Tschacher, 2026), sociology (Weidlich, 1991; Haken, 1999; Weidlich, 1999; Müller, 2026), and even reflections on the nature of humor (Tschacher and Haken, 2023). According to Synergetics, self-organized structure formation proceeds as follows: Initially, a system is in a disordered, macroscopically homogeneous state. When the quantity playing the role of the control parameter changes such that the system reaches a critical point, a separation of time scales occurs. This allows the emerging macro-dynamics to be described by only a small number of slowly varying unstable modes, so-called *order parameters*. The the remaining, stable modes adjust to values dictated by the order parameters almost instantaneously and can thus be approximately eliminated from the description of the system (Haken and Wunderlin, 1991). In synergetic terms, the order parameter *enslaves* the stable modes, and *circular causality* sets in: the stable modes give rise to the macroscopic order parameters, while the order parameters govern the behavior of the stable modes. For example, when applied to synchronization, a highly prominent phenomenon in network physiology, this means that the phases of a population of coupled oscillators collectively constitute an order parameter that, in turn, feeds back onto the individual phase dynamics (Kuramoto, 1975; Schöll, 2022b). Transferred to neural systems, for instance, this would imply that globally coherent spiking patterns somehow govern the firing dynamics of individual neurons.

Explanations invoking enslavement as part of circular causality appeal to some form of downward causation. As already motivated, this invites the question of how enslavement is to be interpreted ontologically: Is the order parameter attributed a real and independent existence, i.e., do such explanations refer to genuine causal forces? Or is it merely a scientifically immensely fruitful description or heuristic device? The philosophical debate on emergence and reductionism offers no consensus on this issue, given the ambivalent status of self-organization theories, which have been taken to support both reductionist and anti-reductionist positions (Kuhlmann, 2009). In what follows, I outline opposing perspectives on the interpretation of enslavement and their compatibility with, or support from, Synergetics.^1^

## 2 Insights from philosophy and philosophy of science

### 2.1 Descriptive vs. causal reading of enslavement

Achim Stephan distinguished between two readings of the slaving principle. In the descriptive reading, taken purely as a mathematical relation, it amounts to nothing more than the thesis that the macroscopic behavior of the system can be *represented* by only a few variables, i.e., the order parameters. By contrast, a stronger causal reading implies that the behavior of the system’s constituents is *determined* by the macroscopic entity represented by the order parameter (Stephan, 2005). Shifting from a descriptive to a causal reading thus transforms a thesis about representation into an ontological claim about the existence of order parameters as causes (Kuhlmann, 2009). Without further argument, such a shift amounts to a *post hoc ergo propter hoc* fallacy (Stephan, 2005).^2^ However well the descriptive thesis may be empirically confirmed, claims about genuine causal relationships require further argumentative support from metaphysics and philosophy of science.

### 2.2 Causation, downward causation, reduction and emergence

Causation is one of the central topics of metaphysics. The contemporary predominant scientific view, i.e., the background conception implicit in much of scientific practice, rests on three intertwined presumptions: causation is efficient (causes produce or bring about their effects), diachronic (causes precede their effects) and transitive (if A causes B and B causes C, then A causes C). As with any standard view, it is not carved in stone but it does place the burden of proof on alternative accounts, at least insofar as they aim to be in line with a broadly naturalistic worldview.

Downward causation is the idea that higher-level properties or systems exert causal influence on their lower-level constituents. This is not to be conflated with ordinary macro-causation, as in cases where a stone breaks a window pane and thereby disrupts molecular bonds. Rather, what is at issue is *reflexive* downward causation: a macro-entity exerts causal influence on the very constituents that realize it. The question of whether downward causation exists, and if so, what it amounts to, is closely intertwined with the debate on reduction and emergence. Reductionists maintain that higher-level phenomena can be reduced, that is, traced back to a more fundamental level. By contrast, proponents of strong emergence hold that there are higher-level phenomena that are genuinely novel and, in principle, irreducible to a more fundamental level. Through downward causation, these emergent macro-phenomena exert causal influence on their lower-level constituents. Strong emergence parallels the conception developed by British emergentist in the 1920s and exhibits a certain tension with a natural scientific worldview. Besides, there are weaker notions of emergence that are compatible with forms of reducibility (Chalmers, 2006; Hoyningen-Huene, 2009; Ellis et al., 2012).

Reductionist claims, just like their anti-reductionist counterparts, can take various forms, that ought to be kept distinct. For example, while *epistemological*, *explanatory* and *methodological reductionism* maintain that knowledge of, and explanations for, higher-level phenomena can be reduced to lower-level knowledge and explanations, or that higher-level phenomena should be studied using lower-level methods, *ontological reductionism* holds that higher-order entities are nothing more than their lower-level constituents, with no interactions or laws playing any role beyond those of the lower level (Hoyningen-Huene, 2009). In considering how circular causality is to be interpreted, the ontological aspect is at issue.

### 2.3 Fundamental objections against downward causation

Much of the criticism on downward causation is associated with Jaegwon Kim’s reflections on the causal efficacy of mental properties, as set out in various exclusion arguments (Walter, 2006; Robb et al., 2023). His arguments can be extended to the question of whether properties of special sciences can causally influence the physical entities on which they depend (Wilson, 2015). Yet, the very idea of downward causation, even in the context of inanimate physical systems, gives rise to fundamental objections.

First, downward causation is criticized as viciously circular. Consider the well-known vortex example Roger W. Sperry gave in his account of consciousness (Sperry, 1969). He argued that a vortex exemplifies downward causation: the circularly moving water molecules give rise to the vortex, which in turn simultaneously causes the water molecules to move in a circle. The problem now arises from the assumption that causation is transitive. Applied to Sperry’s vortex, the motion of the water molecules causes the vortex and the vortex causes the movements of the water molecules. Hence, the motion of the water molecules turns out to be self-causing (Hulswitt, 2006). Kim highlights the apparent paradox by asking whether it is “coherent to suppose that the presence of X is entirely responsible for the occurrence of Y (so Y’s very existence is totally dependent on X) and yet Y somehow manages to exercise causal influence on X?” (Kim, 1999).

A second objection is linked to the presumption of causal closure at the micro-level. Exclusion arguments typically invoke the causal closure of physics with respect to the mental as a premise (Walter, 2006). More generally, causal closure implies that every micro-level effect with a sufficient cause has a sufficient micro-level cause governed by micro-level laws. For macro-level causes to make a difference at the micro-level–and not be merely epiphenomenal or causally overdetermining–they would have to violate and override micro-level laws (Bedau, 2002).

### 2.4 Two exemplary metaphysical embeddings and interpretations of enslavement

In what follows, I outline two contrasting interpretations of enslavement: one embedded in Mark Bedau’s reductionist concept of weak emergence, and the other within the anti-reductionist idea of *downward causation through constraints*.

#### 2.4.1 Enslavement as reducible downward causation: Bedau’s weak emergence

Bedau’s concept of weak emergence links the temporal unfolding of macroscopic phenomena to a distinctive explanatory demand captured by *explanatory incompressibility*: “If P is a macro-property of some system S, then P is weakly emergent if and only if P is generatively explainable from all of S’s prior micro-facts but only in an incompressible way” (Bedau, 2008). Tracing the complex, dynamic micro-processes thus involves an inherently elaborate and protracted explanatory effort. Without presupposing a specific notion of explanation, generative explanations of macro-level phenomena are required to be true, accurate, and complete descriptions of how a system evolves over time, starting from a fully specified initial micro-level state and boundary conditions. An explanation counts as *incompressible* just in case no ‘explanatory shortcut’ can preserve truth, accuracy, and completeness (Bedau, 2008)^3^.

Weak emergence presupposes that the system is *locally reducible*, i.e., that macro-states can, at any given time, be synchronously reduced to the states of their micro-constituents and that micro-dynamics are governed by explicit rules specifying how the state of each micro-entity evolves as a function of its own current state and the states of interacting micro-entities. As a consequence, a micro-causal explanation can, at least in principle, be reconstructed via stepwise numerical simulation, even if this proves infeasible in practice due to excessive dimensionality or incomplete knowledge of initial or boundary conditions. Since Bedau’s account not only accommodates but presupposes ontological and causal reductionism, downward causation turns out to be no more metaphysically problematic than ordinary macro-causation. By virtue of its diachronic character, it avoids circularity, since macro-properties influence micro-conditions only at later times, not at the time of their own instantiation. Likewise, causal closure of the micro-level remains unchallenged, since macro-causes amount to aggregated micro-causes rather than competing with them for causal relevance (Bedau, 2002).

Synergetics is readily situated within this conceptual framework, assuming that the relevant micro-dynamics are completely described by a set of differential equations $\dot{q}=N\left(q,\mu \right)$ that specify the explicit rules according to which the system evolves over time. (Importantly, Bedau’s account also allows for open systems and fluctuations (Bedau, 2002).) Of course, Synergetics does not aim at generative explanations but seeks to identify qualitative, asymptotic patterns in macroscopic behavior in terms of order parameter equations. Their explanatory power derives precisely from abstracting away exhaustive detail, or as Haken put it, “[the] restriction to qualitative, macroscopic changes is the price to be paid in order to find general principles” (Haken, 2006). However, for synergetic self-organization phenomena to qualify as weakly emergent, the crucial condition is that their corresponding generative micro-explanations be incompressible. Order parameter equations, after all, are no explanatory shortcuts in Bedau’s sense. Moreover, the fact that macro-level phenomena admit description by order parameter equations places them within what Bedau terms *robust emergence*: cases in which law-like micro-generalizations arise because macroscopic structures are independent of initial conditions or micro-dynamic details, or are multiply realizable (Bedau, 2002; Bedau, 2008). From this perspective, enslavement amounts to an uncontroversial and metaphysically innocuous form of reducible downward causation.

#### 2.4.2 Taking an anti-reductionist stance: downward causation through constraints

I take *downward causation through constraints* (DCC) as an exemplary anti-reductionist account for two reasons: first, it aligns with a broadly naturalistic view and is therefore more compatible with the natural sciences than concepts of strong emergence and second, its proponents have illustrated it by means of Bénard convection, a paradigm of Synergetics.

DCC emphasizes the role of global or system-wide influences termed *constraints*. Applied to Bénard convection, these include system boundaries, energy exchange with the environment, gravity, volume forces, symmetries and conservation laws. Once the temperature difference exceeds the critical value, Bénard cells arise from the local dynamics of the fluid elements under these constraints and come to play the role of new *internal* constraints that both restrict and enable the dynamics of the constituent fluid elements: macroscopically homogeneous states are no longer accessible, while rotational motion as part of a Bénard cell becomes possible (Bishop, 2008; Bishop 2012).

DCC is embedded in a far-reaching anti-reductionist framework, according to which complex nonlinear systems in principle elude reductionist analysis (Bishop, 2012; Juarrero, 2009; Ellis, 2012). Instead, its proponents advocate *contextual emergence* according to which “[t]he properties and behaviors of a system at a particular [micro-]level (including its laws) offer necessary but not sufficient conditions for the properties and behaviors at a higher level” (Bishop, 2011). Moreover, the properties of the micro-constituents and the laws governing micro-dynamics are not even sufficient to fully determine behavior at the micro-level itself. On this view, fundamental laws of nature are taken merely to delineate a space of possible behaviors without fixing which of these possibilities is actually realized,^4^ thereby leaving a “causal slack” (Ellis, 2010) that must be closed by *contextual conditions* at a higher level (Bishop and Ellis, 2020). In Bénard convection, the relevant context includes the constraints mentioned above and the Bénard structures themselves (Bishop, 2012).

DCC departs from the established scientific view on causation in three respects. First, it invokes the Aristotelian notion of formal cause (Bishop, 2008; Juarrero, 2002). Second, it expands the class of causal relata (Hulswitt, 2006); beyond events or instantiated properties, the DCC view includes symmetries, conservation laws, system geometry, energy flows, volume forces, and relational structures such as Bénard cells among them. And third, causation is conceived as *synchronous*, i.e., at any given time, the motion of the fluid elements is causally influenced by the Bénard cell instantiated at that very same moment (Bishop, 2012).

Against this backdrop, DCC can sidestep the objections raised against downward causation. The charge of self-causation is defused by appealing to formal causation, which, in contrast to efficient causation, does not entail transitivity (Hulswitt, 2006). Thus, DCC goes hand in hand with a non-vicious form of self-causation, as “[the] constraint and maintenance functions of Bénard cells are not absurd in any obvious sense though they act more as formal or structuring causes” (Bishop, 2008). Likewise, the objection from causal closure cannot even be raised against DCC, since rejecting this assumption is part and parcel of the view. Still, the assumption that macroscopic constraints seamlessly fill the purported causal gap without overriding micro-dynamics remains speculative unless further substantiated.

DCC supports a causal reading of circular causality, according to which macroscopic structures represented by order parameters act as internal constraints on their constituents. With view to Bénard convection, Bishop writes: “ [L]arge-scale structures arise out of fluid elements, but they also condition or constrain the contributions the fluid elements can make […] (e.g., due to enslavement […])” (Bishop, 2008). However, DCC and Synergetics differ significantly in their starting premises, as Synergetics does not presuppose a causal underdetermination of the micro-level that needs to be closed, *inter alia*, by macroscopic entities represented by order parameters. Rather, it maintains that the system’s micro-dynamics are fully specified by governing dynamical equations.^5^ Furthermore, DCC has the significant drawback that, as yet, it lacks a metaphysical framework capable of accommodating the coexistence of efficient and formal causes, along with their manifold causal relata. However undisputed the methodological and explanatory advantages of anti-reductionist analyses of complex systems may be, they do not automatically entail greater ontological significance (Kuhlmann, 2009). Thus, considerable metaphysical groundwork remains to be done if DCC is to become a serious competitor to reductionist accounts.

## 3 Discussion

Inter-level causation across scales, especially downward causation, is a topic of ongoing relevance not only within philosophy, but also in complex systems research. Against this background, Synergetics provides a productive interface between philosophical reflection and complex systems research. The concepts of circular causality and enslavement provide highly accessible descriptions of self-organization phenomena, not least because they adopt a non-reductionist perspective that takes into account the way systemic macro-causes feed back onto the system’s constituents. While these concepts are explanatorily and methodologically compelling, caution is called for so as to avoid the presumption that the macroscopic entities represented by order parameters possess independent–that is, irreducible–causal efficacy. Synergetics does not, by itself, establish ontological anti-reductionism, and current debate tends to favor reductionist interpretations. Nevertheless, the debate is far from being settled and further progress on these questions will likely depend on sustained integration of both philosophical reflection and empirically informed perspectives and approaches.

## Data Availability

The original contributions presented in the study are included in the article/supplementary material, further inquiries can be directed to the corresponding author.

## Footnotes

1 I do so from the standpoint of scientifically informed metaphysics, which takes scientific theories and empirical findings as primary sources of evidence for understanding the fundamental nature of reality, while acknowledging philosophy’s distinctive authority in providing analytical and conceptual resources. Against this background, scientific theories are not expected to settle metaphysical debates; rather, they guide them by providing empirical grounding and constraints, and by prompting further metaphysical reflections.

2 Stephan can be read as suggesting that Haken took his causal terminology too literally in a metaphysical sense. I therefore wish to emphasize that Haken actually held a nuanced stance attentive to the underlying philosophical subtleties (see, e.g., Schiepek and Haken (2010)).

3 Although framed in epistemic terms, Bedau ties his definition to the ontological claim that the incompressibility of micro-causal explanations mirrors a genuine feature of the objective micro-causal structure of the world (Bedau, 2008).

4 On this view, for example, General Relativity describes the possible trajectories of an apple falling from a tree, but it does not include the contextual condition of a hand reaching out to catch the apple, which actualizes one of these possibilities.

5 And boundary conditions could, in principle, also be described at the micro-level.
